# Physical Activity During Pregnancy - Past and Present

**DOI:** 10.34763/devperiodmed.20182201.0913

**Published:** 2018-04-12

**Authors:** Miriam Katz

**Affiliations:** 1 Professor Emerita of Obstetrics-Gynecology, Faculty of Health Sciences, Ben-Gurion University of the Negev, Beer-Sheva, Israel

**Keywords:** Physical activity, maternal health, fetal health risks, pregnancy outcome

## Abstract

The possible implications of physical activity during the period of pregnancy have been much debated over recent decades. This brief appraisal integrates knowledge from an array of position papers, systematic reviews, meta-analyses, and recommendations provided by specialty board committees. The medical community is becoming more and more aware of the beneficial effects of mild and moderate physical activity on the mother and the fetus, including improved clinical correlates of subsequent vaginal delivery, as contrasted to the clearly unbeneficial effects of a sedentary lifestyle.

## Introduction

Pregnancy is associated with considerable physiological and psychological changes that may promote sedentary behavior in women. Such behavior is associated with increased risks for gestational diabetes, arterial hypertension and excessive weight gain, all of them jeopardizing maternal and fetal well-being in pregnancy.

Since at present a large proportion of pregnant women engage in regular physical activity during pregnancy, it is imperative that the obstetrician becomes familiar with the effects that physical activity can exert on maternal health and pregnancy outcomes.

The debate with regard to the possible risks and benefits of physical activity during pregnancy goes back to biblical times when Hebrew writers observed that slaves had much easier deliveries than their Egyptian patrons who led a sedentary life [1]. Both Aristotle (4^th^ Century B.C.E.) and later Plutarch of Chaeronea (1^st^/2^nd^ Century C.E.) urged Spartan and other Greek women to exercise in order to decrease the pain of childbearing [2].

In contrast, it is amazing that by the end of the 19^th^ century pregnant women were encouraged to remain indoors, keep themselves “confined”, and advised to avoid exercise since “during gymnastics there was a risk that the female organs might slip when straddling and female fertility may be at severe risk“ [2].

During the 20^th^ century, the advances in knowledge of anatomy and physiology led to a dramatic change in the approach to physical activity in general and during pregnancy in particular. Already in the 1930’s antenatal exercise classes taught by Heardman, a physiotherapist, were introduced in England and Sweden [3]. In 1949, the U.S. Children’s Bureau published their recommendation for prenatal exercise stating that, in cases without maternal complications, pregnant women may continue their typical activities, such as home duties or gardening on a daily basis. In 1959, Karmel and Bing, also in the United States, popularized the Lamaze method that focused on physiological and psychological preparation for childbirth [4]. The Lamaze method is still very popular in many medical centers today.

In 2015, the American College of Obstetricians and Gynecologists published a Committee Opinion with regard to physical activity and exercise during pregnancy and the postpartum period. The Committee recommended that pregnant women with no risk factors should exercise 20-30 minutes per day engaging in aerobic and strength-conditioning exercise before, during, and after pregnancy [5].

Overall, the last 100 years witnessed a great interest in this subject as evidenced by an enormous bulk of literature published in the most prestigious medical journals. Below, we present a personal selection of some of these reports.

## Physiological aspects

Szumilewicz *et al*. quote the U.S. Department of Health and Human Services definition of “physical activity” that it is “defined as any bodily movement produced by the contraction of skeletal muscle that increases energy expenditure above a basal level”. In contrast, “exercise” represents “a form of physical activity that is planned, structured, repetitive, and performed with the goal of improving health or fitness” [6].

The results of a detailed literature review on the physiology of exercise in pregnancy were, among others, that [7]:

– maximal aerobic power is well-preserved in pregnant women who remain physically active;
– in contrast, anaerobic working capacity may be reduced in late gestation;
– responses to prolonged submaximal exercise (>30 minutes) in late gestation include a moderate reduction in maternal blood glucose concentration, which may transiently reduce fetal glucose availability;
– the normal response to sustained submaximal (maternal) exercise is an increase in fetal heart rate (FHR) baseline;
– transient reductions in FHR reactivity, fetal breathing movements, and FHR variability may also occur in association with more strenuous exercise.

Consequently, Wolfe and Weissgerber stated that healthy women undergoing normal pregnancies can participate safely in moderate prenatal fitness programs and maintain or improve physical fitness without harming fetal growth and development [7].

During pregnancy, sustained exercise sessions cause an intermittent reduction in oxygen and substrate delivery to the interphase that may exceed 50% during the exercise, however, regular bouts of sustained exercise or exercise training probably improve oxygen and substrate delivery to the fetus at rest [8].

As to work effciency, the energy cost of standard submaximal exercise in pregnancy depends on whether the activity is weight-bearing or weight-supported. For weight-supported exercise (such as cycling), the energy requirement above resting metabolic rate is not significantly affected by pregnancy or advancing gestational age. In contrast, for weight-bearing activities (such as walking or jogging), the energy requirement increases in proportion to maternal weight gain [7].

Swimming has been advocated as an optimal exercise during pregnancy due to its buoyant effects and the thermally conductive properties of water [9]. Stationary upright cycling for 30 minutes at a maternal heart rate around 140 beats per minute (bpm) or exercising for 15 minutes at a rate of 155 bpm has no negative effect on the woman or the fetus. Also walking is a recommended activity. In contrast, extreme sports such as scuba diving, high-altitude exercise including skiing, hiking, mountain biking, and running, as well as sports with abdominal trauma risk from contact or falling are discouraged. The list of the latter activities also includes downhill skiing, waterskiing, horseback riding, road cycling, surfing, basketball, racquet sports, ice hockey, soccer, and gymnastics [6, 9, 10]. Definitely, both prudence and moderation are a good general advice. Interestingly, a recent careful testing of maternal and fetal responses to 26 yoga postures confirmed that yoga is very safe not only for the mother, but also for the baby [11]. Antenatal pelvic floor muscle exercises are gaining in popularity and there are essentially no harmful effects ascribed to them, either, whereas they may prevent urinary incontinence in late pregnancy and definitely prevent urinary incontinence in the postnatal period [12].

From a systematic review, the exercise intensity prescription should be mild to moderate for previously sedentary women, and moderate to high for previously active women [10]. Thus, moderate intensity activity can always be a good advice of the obstetrician for gravidae without contraindications to exercise. These recommendations are based on the current knowledge of the impact of moderate-intensity, low-impact, aerobic exercise performed by the pregnant woman at least three times a week and thus they may be subject to broadening, rather than narrowing, as further evidence accumulates. Let us cite here the Jukic *et al*. data [13] where it was shown in a prospective study that vigorous recreational activity does not appear to be detrimental to the timing of birth or to birthweight. In line, in a carefully controlled study, inactive, regularly active, and highly active pregnant women were subjected to one or two 30-minute treadmill sessions. Umbilical artery Doppler indices were similar pre-exercise and postexercise among groups. Moreover, postexercise FHR tracings met the criteria for reactivity within 20 minutes after all the tests [14], consistent with the lack of acute aggravation in fetal well-being.

Absolute and relative medical contraindications to exercise during pregnancy are given in Table I. Overall, any significant obstetric or other medical conditions should prevent from engagement into unsupervised vivid physical activity. As evidenced in a survey, approximately half of the American obstetricians recommended a reduction in exercise load during the third trimester [15], possibly as a preventive measure.

**Table I:** Guidelines regarding absolute and relative contraindications to exercise during pregnancy. Adapted from Olson *et al*. [9].

Absolute Contraindications	Relative Contraindications
Incompetent uterine cervix Intrauterine growth restriction	Extreme underweight (body mass index < 12.0) or maternal eating disorder (such as anorexia nervosa and bulimia)
Multiple gestations (≥ triplets)	Maternal cardiac arrhythmia or cardiovascular disorder
Persistent second or third trimester bleeding	Mild to moderate respiratory disorder
Placenta previa after 25–28 weeks of gestation	Previous spontaneous abortion
Preeclampsia	Severe anemia (Hemoglobin concentration < 100 g x L^-1^)
Pregnancy-induced hypertension	Twin pregnancy after 28 weeks of gestation
Premature labor during current or prior pregnancy	Poorly controlled hypertension
Premature rupture of membranes	Poorly controlled type 1 diabetes
Risk of premature labor	History of extreme sedentary lifestyle
	Orthopedic limitations
	Extreme morbid obesity
	Poorly controlled seizure disorder or hypothyroidism
	Other significant medical condition

## Clinical aspects

Since it seems possible to improve pregnancy outcomes in both healthy, low-risk women and a variety of high-risk populaces by simply modifying maternal physical activity and dietary carbohydrate intake during pregnancy [8], this notion sparked an increased interest in maternal exercises. The goals of physical activity during pregnancy include maintaining maternal well-being and establishing a pattern of regular activity that will ultimately prevent the onset of chronic disease associated with a sedentary lifestyle. The main benefits of physical activity in pregnancy are as follows: it helps control weight gain, improves blood circulation (including less swelling), reduces the risks of developing preeclampsia and gestational diabetes, and increases the chances for a shorter spontaneous vaginal delivery. As for the baby, physical activity normalizes maternal blood glucose and reduces insulin resistance, thus affecting fetal size. Moreover, placental blood flow and nutrient delivery to the developing fetus become stabilized [8, 16].

The majority of women who gained excessive weight are at risk of developing gestational diabetes and preeclampsia, and of having macrosomic babies, all of which can lead to increased complications in labor and delivery. Furthermore, these big babies born to overweight mothers are at a significant risk for developing type 2 diabetes later in life. Thus, it is of importance that in their robust meta-analysis which included 442 studies addressing the effect of exercise on pregnancy, Tobias *et al*. [17] concluded that exercise significantly reduces the odds for the development of gestational diabetes. A review of suggested recommendations for prevention and treatment of gestational diabetes by means of exercise has recently been published [18]. In a Canadian prospective cohort study, physical activity in the first half of pregnancy significantly decreased the occurrence of fetal macrosomia without affecting preterm birth or low birth weight [19].

Other pregnancy-related complications which may seriously jeopardize the pregnancy outcome are hypertensive disorders. They affect about 3-9% of all pregnancies worldwide, with preeclampsia diagnosed in 2-4% of them. Even today hypertensive disorders are one of the main causes of maternal and fetal morbidity and mortality [20]. Therefore, it is important to note that physical activity prior to and during pregnancy has been found to protect the gravidae and parturients from hypertensive disorders by controlling their weight gain, lowering the blood pressure, improving maternal lipid profile, reducing the oxidative stress and inflammation, as well as improving placental growth and vasculature [8]. A group of Scandinavian researchers reported a significantly lower incidence of elevated blood pressure in a group of 59 573 prospective mothers who exercised more than 25 times a month starting from 16 weeks of pregnancy [21].

Regarding other central clinical correlates, one of the main fears of the pregnant patient is that physical activity may increase her risk for miscarriage or premature delivery. Most studies addressing this question state that in a patient with no risk factors (i.e., no previous premature labor, cervical incompetence, etc.) physical activity is safe. However, one should advise against exertion (high-intensity and long-duration exercise) in early pregnancy, especially at 8-12 weeks. In contrast, some already cited data support a reduced risk of preterm birth with vigorous recreational activity, particularly with increased frequency of recreational type activity [13]. In a large Dutch-British population-based cohort study on daily physical activity, 11 759 singleton pregnancies were assessed and no association between physical effort and duration of gestation or fetal survival was found [22].

In a retrospective study, Bungum *et al*. [23] found that nulliparous women exercising during pregnancy had substantially increased odds for a normal vaginal delivery and that such subjects had a lower chance of Cesarean section delivery. Impressively, for those sedentary during pregnancy, the odds ratio for Cesarean delivery was found to be as high as 4.48 [23].

The results of a meta-analysis of observational studies suggested an inverted U-shaped association between physical activity and birth weight [24]. In other words, both extremes in physical activity of the pregnant woman are unbeneficial.

There are undoubtedly many advantages of exercise during pregnancy and therefore, after excluding medical and pregnancy-related risk factors, obstetricians should strongly recommend to their patients to be physically active. Non-extreme physical activity prior to and during pregnancy is not only safe for fetal health but is also associated with numerous maternal health benefits, including prevention and better control of gestational diabetes, excessive weight gain, and increased blood pressure. It is worth mentioning that exercise also reduces lower back pain and has positive effects on both maternal mental health and quality of life.

## Conclusions

The recent recommendations from the American College of Obstetricians and Gynecologists clearly embrace the research cited above. They can be summarized as follows [5]:

1. physical activity in pregnancy has minimal risks and has been shown to benefit most women, although some modification to exercise routines may be necessary because of normal anatomic and physiologic changes and fetal requirements;
2. a thorough clinical evaluation is necessary before recommending an exercise program to ensure that a patient does not have medical reasons to avoid exercise;
3. women with uncomplicated pregnancies should be encouraged to engage in both aerobic and strength-conditioning exercises before, during, and after pregnancy;
4. obstetrician–gynecologists and other obstetric care providers should carefully evaluate women with medical or obstetric complications before making recommendations on their physical activity participation during pregnancy;
5. regular physical activity during pregnancy improves or maintains physical fitness, helps with weight management, reduces the risk of gestational diabetes in obese women, and enhances psychological well-being.

This statement testifies to a tremendous shift in both medical thinking and practical approach from the necessarily conservative position of early guidelines because of lack of research decades ago to the current active encouragement of pregnant women to do mild and moderate exercise as a physiological means to maintain a healthy, or healthier, pregnancy. What a spectacular change we are witnessing!
